# Gut microbiota research nexus: One Health relationship between human, animal, and environmental resistomes

**DOI:** 10.1002/mlf2.12101

**Published:** 2023-12-26

**Authors:** Yuhao Fu, Qingyuan Dou, Kornelia Smalla, Yu Wang, Timothy A. Johnson, Kristian K. Brandt, Zhi Mei, Maoyuan Liao, Syed A. Hashsham, Andreas Schäffer, Hauke Smidt, Tong Zhang, Hui Li, Robert Stedtfeld, Hongjie Sheng, Benli Chai, Marko Virta, Xin Jiang, Fang Wang, Yong‐Guan Zhu, James M. Tiedje

**Affiliations:** ^1^ State Key Laboratory of Soil and Sustainable Agriculture, Institute of Soil Science Chinese Academy of Sciences Nanjing China; ^2^ University of Chinese Academy of Sciences Beijing China; ^3^ Julius Kühn Institute (JKI) Federal Research Centre for Cultivated Plants Braunschweig Germany; ^4^ Department of Animal Sciences Purdue University West Lafayette Indiana USA; ^5^ Section for Microbial Ecology and Biotechnology, Department of Plant and Environmental Sciences University of Copenhagen Frederiksberg C Denmark; ^6^ Sino‐Danish Center (SDC) Beijing China; ^7^ Department of Microbiology University of Helsinki Helsinki Finland; ^8^ Department of Plant, Soil and Microbial Sciences, Center for Microbial Ecology Michigan State University Michigan USA; ^9^ Department of Civil and Environmental Engineering Michigan State University Michigan USA; ^10^ Institute for Environmental Research RWTH Aachen University Aachen Germany; ^11^ Laboratory of Microbiology Wageningen University & Research Wageningen The Netherlands; ^12^ Environmental Microbiome Engineering and Biotechnology Laboratory, Center for Environmental Engineering Research, Department of Civil Engineering The University of Hong Kong Pokfulam Hong Kong China; ^13^ Institute of Agricultural Resources and Environment Jiangsu Academy of Agricultural Sciences Nanjing China; ^14^ Key Laboratory of Urban Environment and Health, Institute of Urban Environment Chinese Academy of Sciences Xiamen China; ^15^ State Key Laboratory of Urban and Regional Ecology Chinese Academy of Sciences Beijing China

**Keywords:** antibiotic, antimicrobial resistance, gut microbiota, modulatory strategies, One Health

## Abstract

The emergence and rapid spread of antimicrobial resistance is of global public health concern. The gut microbiota harboring diverse commensal and opportunistic bacteria that can acquire resistance via horizontal and vertical gene transfers is considered an important reservoir and sink of antibiotic resistance genes (ARGs). In this review, we describe the reservoirs of gut ARGs and their dynamics in both animals and humans, use the One Health perspective to track the transmission of ARG‐containing bacteria between humans, animals, and the environment, and assess the impact of antimicrobial resistance on human health and socioeconomic development. The gut resistome can evolve in an environment subject to various selective pressures, including antibiotic administration and environmental and lifestyle factors (e.g., diet, age, gender, and living conditions), and interventions through probiotics. Strategies to reduce the abundance of clinically relevant antibiotic‐resistant bacteria and their resistance determinants in various environmental niches are needed to ensure the mitigation of acquired antibiotic resistance. With the help of effective measures taken at the national, local, personal, and intestinal management, it will also result in preventing or minimizing the spread of infectious diseases. This review aims to improve our understanding of the correlations between intestinal microbiota and antimicrobial resistance and provide a basis for the development of management strategies to mitigate the antimicrobial resistance crisis.

## INTRODUCTION

Antibiotics, extensively utilized in clinical infection treatment and as plant and animal husbandry growth promoters, have significantly contributed to increased life expectancy and socioeconomic progress^1^. A spatial modeling study found a 46% increase in global antibiotic consumption since 2000, with a large proportion of the prescriptions being deemed inappropriate, particularly in low‐ and middle‐income countries (LMICs)^2^. Though intrinsic resistance existed in nature long before the anthropogenic use of antibiotics^3^, the rampant use of antibiotics has accelerated the spread of antibiotic resistance genes (ARGs) at a rate surpassing natural evolution, thereby posing a rising global health concern^4^, ^5^. Recent global studies estimate that antimicrobial resistance was linked to approximately 4.95 million deaths in 2019 across 204 countries and territories, including 1.27 million deaths directly attributable to antibiotic‐resistant infections^6^. ARGs, unlike traditional chemical and biological pollutants, can be passed to progeny via vertical gene transfer or spread by horizontal gene transfer between different pathogenic and commensal species, even those with no apparent taxonomic relationships, thereby overcoming conventional mating barriers^7^. The mechanisms of horizontal gene transfer, including conjugation, transformation, transduction, and vesiduction, along with vertical gene transfer, contribute to the evolution and global spread of ARGs in microbial ecosystems. Notably, the transmission events of ARGs are particularly high in diverse microbial environments, such as animal manure^8^, wastewater treatment plants^9^, and the guts of animals and humans^10^, ^11^, ^12^.

The gut microbiota, comprised of thousands of diverse species colonizing the animal/human intestinal tract, often maintain symbiotic relationships with their hosts^13^, ^14^. With essential functions such as providing dietary nutrients, protecting against opportunistic pathogens, and shaping the immune system, gut bacteria are crucial to host health^15^. Undoubtedly, perturbations in the stability of the gut microbiota, such as the loss of specific taxonomic groups or a decrease in microbial diversity, can adversely impact host health^16^. The gut microbiota has recently attracted attention as a reservoir of ARGs^17^. Specifically, the gut microbiota harbors a diverse array of ARGs conferring resistance to antibiotics, such as aminoglycosides, beta‐lactams, glycopeptides, and tetracyclines, displaying varying abundances among individuals^18^. Orally applied antibiotics can markedly alter the taxonomic and functional compositions of the gut microbiota, selecting for and facilitating colonization by antibiotic‐resistant bacteria (ARB) and accumulation of ARGs^19^. Such dysbiosis can amplify the burden of ARGs, potentially reducing drug efficacy and promoting the invasion of ARG‐carrying pathogenic and nonpathogenic bacteria into the bloodstream, immune system, and other organ systems^16^. Moreover, the proliferation of ARB in the gut can be influenced by multiple factors, including disease, inflammation, diet, probiotics, soil/water/air environmental exposure, and many others^20^. Given the pathways of antimicrobial resistance dissemination, ARGs present in gut microbiomes can be exchanged between the gut bacteria and introduced to exogenous bacteria, even those transiently passing through the intestine^21^. The extensive use of antibiotics in agricultural practices promotes the evolution of ARG‐encoding microorganisms in animal guts, turning animal farms into hot spots for ARG dissemination^22^. Subsequently, unmetabolized antibiotics and ARG‐containing bacteria excreted in urine and feces from animals or humans are often deposited in environmental reservoirs^23^. In turn, various transmission pathways enable the environmental resistome to spread to humans or animals, including direct contact and food chains^24^. These interconnected processes underscore the complex relationships among humans, animals, and the environment under the One Health framework, with the gut microbiota being a crucial link in these transmission processes.

A comprehensive understanding of the fate, transfer, and dynamics of ARGs is urgently needed to propose effective strategies to reduce the spread of ARGs or to mitigate the risks of antimicrobial resistance. Accordingly, this review first provides an overview of animal and human gut resistomes, encompassing the source, transmission, and potential implications of gut ARGs. It then examines several factors, including diet, age, gender, living conditions, and probiotics, affecting gut resistome profiles. Subsequent sections recommend effective strategies at the national, local, personal, and microbial levels to combat antimicrobial resistance and enhance the stability and resilience of the gut microbiome. Finally, it addresses the challenges and prospects of managing antimicrobial resistance.

## ARGs IN ANIMAL GUT MICROBIOMES

### The gut microbiome as a reservoir for antimicrobial resistance

The emergence and spread of ARGs through the gut microbiome of various animals—including livestock, pets, and wildlife—play a pivotal role in the dissemination of antimicrobial resistance^25^, ^26^. In livestock farming and aquaculture, veterinary antibiotics are routinely used to treat bacterial diseases and also can be added to animal feeds as growth promoters and prophylactic drugs to increase farm production and profit^22^. This practice, however, inadvertently selects for bacteria carrying ARGs, thus contributing to the proliferation of ARGs in animal gut microbiomes^27^, ^28^. For instance, the administration of either amoxicillin or thiamphenicol was found to enhance the relative abundance of ARGs conferring resistance to phenicols and beta‐lactams in the gut microbiome of chickens^29^. Similarly, in‐feed antibiotics, including chlortetracycline, sulfamethazine, and penicillin, resulted in the enrichment of 23 ARGs associated with sulfonamide and aminoglycoside resistance in swine gut microbiota compared to nonmedicated groups^30^. Heavy metals, such as copper and zinc, are frequently used as feed additives in livestock production due to their positive impact on animal growth and health, particularly as alternatives to antibiotics^31^. However, these metals can induce co‐selection of antimicrobial resistance due to genetic linkage between heavy metal resistance genes and ARGs (co‐resistance) or physiological collaboration against both antibiotics and metals (cross‐resistance)^32^. For example, feeding pigs with high, but realistic, levels of dietary zinc (2103 mg kg^−1^) was associated with a higher prevalence of multi‐resistant *Escherichia coli* in the swine gut, suggesting a connection between heavy metal exposure and antimicrobial resistance^33^, whereas effects of dietary copper on the gut bacterial resistome may be less clear even at a copper concentration level exceeding the maximum permitted level for pig diets in the European Union as indicated by two recent microbiome studies from a controlled feeding trial^34^, ^35^. Companion animals, specifically cats and dogs, are often overlooked sources of ARGs. Their close contact with both the environment and humans, combined with the frequent administration of antibiotics without veterinary prescriptions, has led to the proliferation of abundant ARGs in their gut microbiome^36^. A study investigating the characteristics of extended‐spectrum beta‐lactamase‐producing *E. coli* isolates from cats and dogs from 2012 to 2017 found that strains resistant to more than three antibiotic classes (multidrug resistance) increased from 67% to 75%, while resistance to six to nine antibiotics increased from 27% to 61%^37^.

Unlike domestic animals, wild animals are not directly treated with antibiotics; instead, their habitats are the primary sources of ARGs in their gut microbiomes. Upon consumption of contaminated water, soil, or organisms, bacteria‐carrying ARGs can be introduced into the gut microbiome of wildlife. For instance, *E. coli* isolated from free‐living small mammals exhibited a prevalence of resistance against four antibiotics in 79% of coastal and 35% of inland individuals^38^, reflecting the spatial variation of antimicrobial resistance in wild animals relative to the distance from human‐impacted water bodies. The higher abundance of ARGs in coastal individuals compared to inland individuals might be related to the direct introduction of bacteria from multiple origins into the aquatic environment and the subsequent contamination of the terrestrial environment. It is noteworthy that even wild animals in remote and pristine areas, such as the polar regions, can acquire antibiotic resistance^39^. Additionally, captive animals, such as pandas, chimpanzees, and gorillas, were found to have a much higher diversity of resistance genes in their gut microbiomes compared to their wild counterparts^40^, ^41^. Combining the analysis of diets, antibiotic exposure, and geography, the perturbation of gut microbiomes and resistomes might be mainly molded by the diet and degree of antibiotic exposure rather than geographical location. Previous research has identified the long‐distance transmission of ARGs by migratory birds, which acquired ARB from human sources and disseminated these through fecal contamination^42^, ^43^. In summary, the gut microbiome in animals represents a significant reservoir of antimicrobial resistance. Recognizing the role of the animal gut in harboring and disseminating ARGs is crucial to fully understand the mechanisms and dynamics of the impact of ARGs on environmental organisms and humans.

### The role of animals in spreading antimicrobial resistance to humans and the environment: One Health perspective

**Figure 1:**
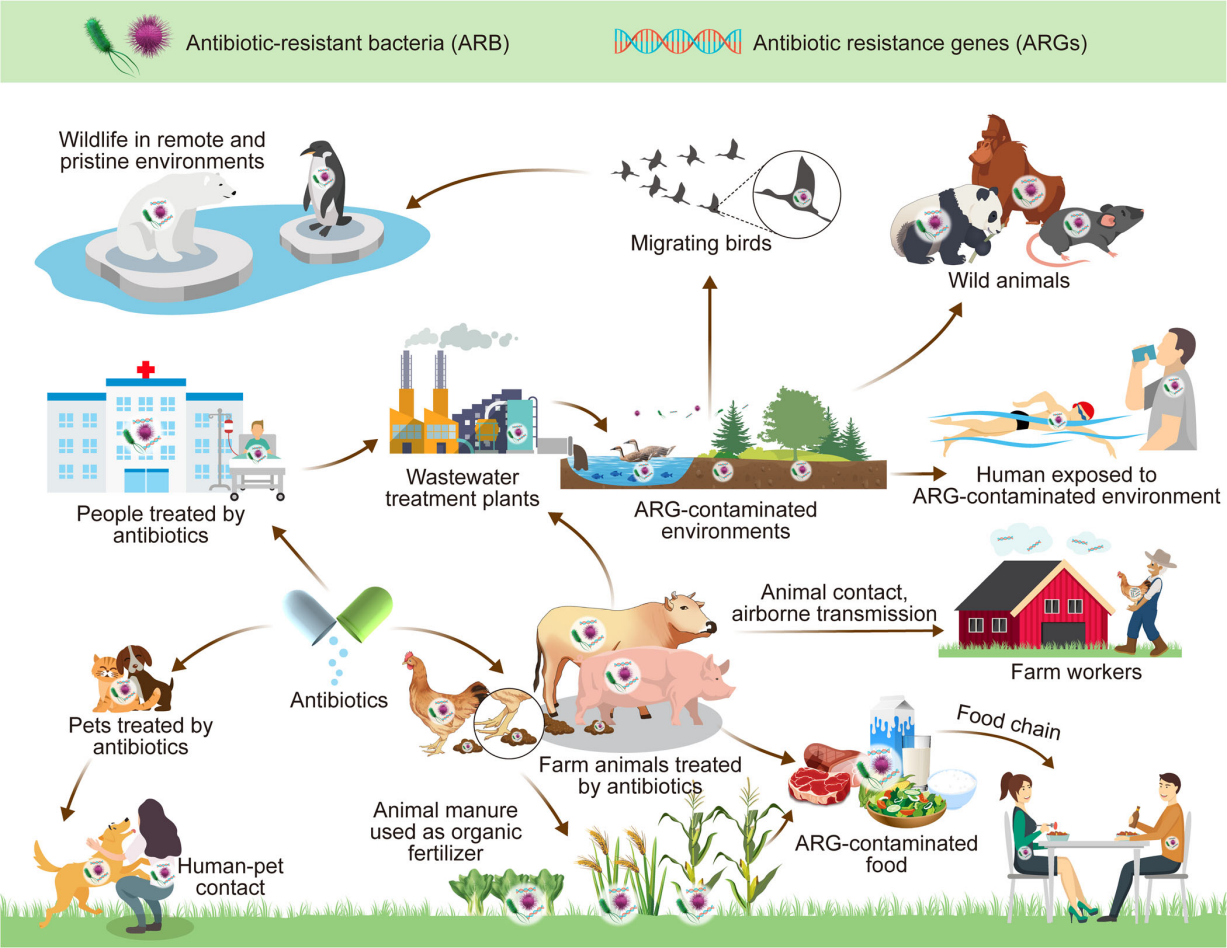
Transmission of ARGs and ARB in the animals, humans, and environment.

Indeed, the shared environment between humans and animals facilitates the exchange of microorganisms and zoonotic pathogens (Figure 1). With over 60% of known infectious diseases in humans being sourced from animals, and more than 40% of emerging infectious diseases in humans being zoonotic, the transmission of ARB from animals to humans represents a significant public health concern (https://www.cdc.gov/onehealth/basics/zoonotic-diseases.html)^44^. Plant‐associated microorganisms also represent an important link by which humans are exposed to pathogenic bacteria, ARB, and ARGs^45^. Direct contact with animals and indirect exposure through the consumption of food products from antibiotic‐contaminated animals/plants contribute to the risk of antibiotic‐resistant infections in humans^46^. Quantitative estimations have shown that food, companion animals, and farm animals (nonoccupational contact) account for 18.9%, 7.9%, and 3.6%, respectively, of carriers of extended‐spectrum beta‐lactamase (ESBL)‐producing *E. coli* and plasmid‐mediated *AmpC*‐producing *E. coli* ^47^. Animal‐derived foods carrying ARB, including milk, eggs, meat, and fat, pose a significant risk of transmission to humans through the daily diet, supported by evidence of the transfer of antimicrobial resistance from veterinary sectors to humans. For instance, the *mcr‐1* gene carriage in *E. coli* isolates, which was observed in 15% of raw meat samples and 1% of inpatient samples, suggested that *mcr‐1*‐mediated resistance originated in animals and was quickly transferred to other microorganisms, including human pathogenic bacteria^48^. A clear example is the outbreak caused by raw milk contaminated with multiple‐antimicrobial‐resistant *Salmonella enterica* ser. Typhimurium in Arizona, USA, where 43% of the *S. enterica* ser. Typhimurium isolates were resistant to chloramphenicol^49^.

Moreover, the environment significantly contributes to the transmission of antimicrobial resistance, acting as a vehicle that connects humans and animals. The “One Health” concept emphasizes the cyclical flow of ARGs across the human, animal, and environmental microbiomes, which is crucial in understanding ARG dynamics^25^, ^26^. The accumulation of antibiotics largely contributed to the development and dissemination of ARGs in source water, drinking water treatment plants, and drinking water distribution systems^50^. The detected ARG concentrations in source water in East China ranged from 1.5 × 10^4^ to 6.4 × 10^5^ copies/ml, in which some specific genes such as *sul* genes are up to 2.4 × 10^5^ copies/ml in the drinking water treatment plants after long‐distance transportation^51^, ^52^. More alarmingly, ARGs significantly increased during pipeline transportation from 1.1 × 10^7^ copies/ml in finished water to 5.1 × 10^8^ copies/ml in tap water, which poses a severe risk of human exposure to such polluted drinking water^53^. Animal manure, often containing diverse antibiotics and their metabolites, is considered valuable organic fertilizers but might have contributed to the enhancement of soil microbiome resistance profiles^54^. Moreover, treated wastewater discharged into the environment carries a residual load of antibiotics and other pharmaceuticals, ARGs, and ARB, thereby augmenting the resistance gene pool in various ecosystems^55^. Bioaerosols are suspended particles that may carry a substantial amount of ARB released from wastewater treatment plants, livestock farms, and medical waste, and drive the global dissemination of ARGs, ultimately threatening human health through intake or inhalation^56^. Humans and animals may be infected by ARB/ARGs through contacting with such contaminated environments or biological materials, such as urine, feces, and blood, in a direct route and the daily ingestion of contaminated food or derived food products in an indirect route^57^. Among those exposure pathways, horizontal gene transfer may largely increase the ARG transmissibility between multidrug‐resistant pathogens and environmental bacteria via mobile genetic element (MGE)‐mediated resistance exchange, thereby showing a clear risk to human health^58^.

## IMPLICATIONS OF ANTIMICROBIAL RESISTANCE ON HUMAN HEALTH

### Relationship between gut antibiotic resistomes and disease

Early‐life antibiotic exposure can profoundly modify the composition and function of the gut microbiome, potentially leading to an increased antimicrobial resistance load, even diseases of autism spectrum disorders^59^, diabetes, cirrhosis, and chronic kidney disease^60^ (Table 1). Newborns are at high risk of acquiring fatal infections due to the immature immune system, and their treatment mainly relies on functioning antibiotics^66^. Antibiotic use would affect the microbiome of infants by promoting the growth of resistant species and suppressing the growth of susceptible species. On the other hand, infants have a higher abundance of ARG‐containing bacteria in their gut microbiota than adults, and the mother's microbiota^66^ and diet^67^ may impact the ARG type and abundance even when not directly exposed to antibiotics. Gut microbes play a pivotal role in the bidirectional communication between the brain and the gut, influencing normal brain functions through various pathways. Disruption of the gut microbiota by early‐life exposure to antibiotics could affect the normal functioning of the central nervous system, which may further negatively impact the epigenetic programming of the child's brain, inducing the emergence of autism spectrum disorder symptoms and an increase of ARGs in the gut resistomes^61^, ^68^. A study involving 1200 middle‐aged and older adults discovered significant changes in the structure of intestinal antimicrobial resistance groups across healthy, prediabetic, and diabetic populations^62^.

**Table 1:** Disease‐associated gut ARGs and effects on disease.

Disease type	Related ARGs	Effects on disease	Reference
Autism spectrum disorder	Abundance of the genes *aac(6′)‐aph(2″), cepA‐49* and *tet(40)* was significantly increased	Serving as markers of autism spectrum disorder	[61]
Diabetes	25 ARGs associated with type 2 diabetes, including *Vancomycin_vanX*, *Multidrug_emrE*, *MLS_ermX*, and *Quinolone_norB*	Positively correlated with the risk of type 2 diabetes	[62]
Cirrhosis	ARGs associated with beta‐lactamase, vancomycin, and quinolones were higher	Disease progression	[63]
Chronic kidney disease	Antibiotic and metal(loid) resistance gene markers: *cadA* and *arsC*	Intestinal transit alterations, decreased protein absorption, and consumption of dietary fiber	[64]
Cardiovascular disease	Genes associated with general intestinal lipid metabolism were increased by antibiotics, such as *Nr1h3*, *Srebf1*, and *Cd36*	Exacerbating serum cholesterol may impact cardiovascular disease	[65]
Hepatic encephalopathy	Much high ARG abundances of beta‐lactamase, vancomycin, RbpA bacterial RNA polymerase binding protein	Disease progression	[63]
Ascites	ARGs higher in aminoglycoside (*ANT* and *APH*), sulfonamide resistance, and selected beta‐lactamases (*SHV*, *CTX‐M*, and *SRT*)	Disease progression	[63]

Patients with cirrhosis are susceptible to infections, which might be related to the altered microbiota composition in their gut, especially with antibiotic‐resistant microorganisms. The abundance of ARGs associated with beta‐lactamase, vancomycin, and quinolone resistance in the gut of cirrhotic patients was higher compared to healthy controls and increased as the disease worsened^63^. These might be due to exposure to healthcare conditions, as they had been hospitalized and/or exposed to antibiotics during the past 6 months. In a comparison of microbial and ARG changes between chronic kidney disease that excluded recent antibiotic use and diseases with major nonrenal and cirrhosis, a great number of pathobionts (e.g., *Acinetobacter*, *Enterobacter*, *Klebsiella pneumoniae*, and *Legionella*) were higher in chronic kidney disease, whereas cirrhotic patients had a higher prevalence of *Escherichia*, *Enterococcus*, *Klebsiella oxytoca*, *Staphylococcus*, and *Streptomyces* spp. Reflecting these, beta‐lactamase resistance genes were present in both disease groups, but cephalosporins, glycopeptide, rifamycin, and vancomycin resistance genes were higher in the gut microbiome of cirrhosis patients^63^. Furthermore, the gut microbiome's importance extends to the gut‐brain axis and distant organs^69^, and high α‐diversity indices of gut ARGs have been found to correlate to high cardiovascular disease and hepatic encephalopathy incidences^62^, ^70^.

### Socioeconomic implications of antimicrobial resistance

The escalating levels of bacterial resistance to antibiotics pose a substantial threat to advancements in both the clinical and agricultural sectors. The threat extends beyond just human health, leading to extensive societal and economic burdens. For the United States, the total economic burden of antimicrobial resistance on the healthcare sector is estimated to be around $20 billion per year. In addition, lost productivity due to the antimicrobial resistance crisis is approximated at about $35 billion a year^71^. The impact is even more devastating in LMICs, where antimicrobial resistance correlates with higher mortality risks and escalated economic costs. The cost of healthcare for patients in LMICs combating antibiotic‐resistant ESKAPE (*Enterococcus faecium*, *Staphylococcus aureus*, *K. pneumoniae*, *Acinetobacter baumannii*, *Pseudomonas aeruginosa*, and *Enterobacter* species) bacteria was found to be significantly higher compared to control groups ($8107 vs. $5469)^72^. Antimicrobial resistance is deepening the poverty in LMICs, primarily due to longer hospital stays, increased treatment costs, and premature deaths, all of which directly affect the total productivity^73^. The World Bank projects that by 2050, antimicrobial resistance will significantly increase extreme poverty levels, and annual global GDP will decrease by 1.1% in the low‐impact antimicrobial resistance scenario and by 3.8% in the high‐impact scenario. LMICs would bear the brunt of these losses^74^. The vast majority (26.2 million) of the additional 28.3 million people who will fall into extreme poverty by 2050 will live in LMICs^74^. An increase in antimicrobial resistance may negatively impact the production and trade of livestock, leading to higher protein prices due to a decrease in protein sources like milk, eggs, and meat^75^. The adverse effects of antimicrobial resistance on livestock production may clash with the demand for animal protein, leading to potential protein shortages. The emergence and spread of antimicrobial resistance are also influenced by factors, such as socioeconomic growth and antibiotic misuse. This phenomenon explains the interplay between the economy, infectious diseases, and self‐medication, thus presenting the occurrence of antimicrobial resistance as a self‐reinforcing cycle in the population^76^.

## THE ROLE OF ENVIRONMENTAL AND LIFESTYLE FACTORS

**Figure 2:**
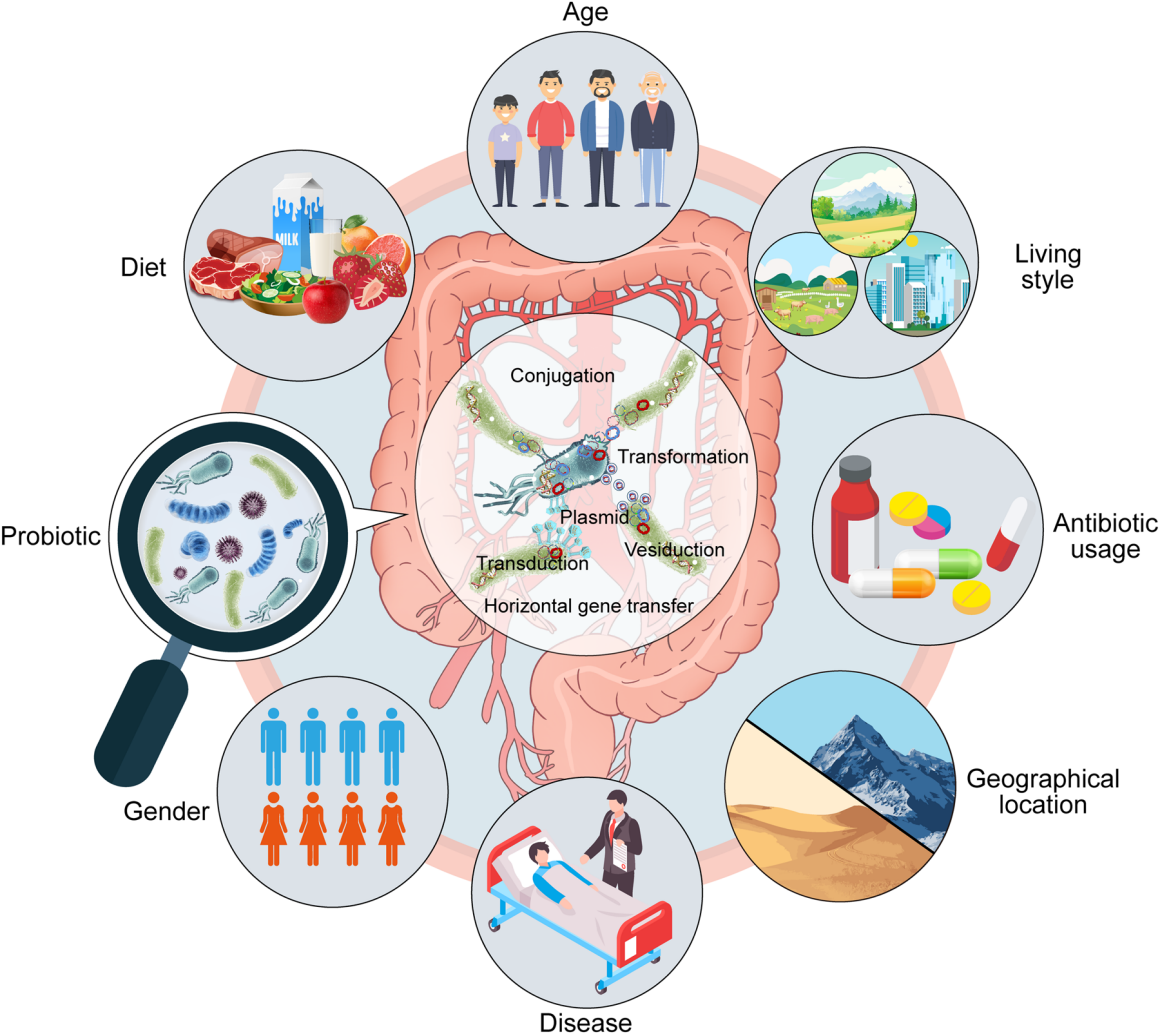
Factors affecting the patterns of antibiotic resistome in gut microbiome.

The spread of ARGs in the gut microbiome is largely determined by factors, such as antibiotic usage, living conditions, disease, age, gender, diet, geographic location, and gut probiotics (Figure 2). An in‐depth analysis of the correlation between these factors and the gut resistome could potentially provide ways to mitigate the extent of antimicrobial resistance.

### Impact of diet on the emergence of ARGs

Different dietary habits can lead to various accumulation patterns of ARGs in the gut resistome. Studies have found that the total abundance of ARGs in the gut microbiome was significantly lower in vegans compared to vegetarians and omnivores (*p* = 0.01)^77^. People with a lower abundance of ARGs in the gut consume significantly more fiber compared to those with medium and high abundance of ARGs^78^. This might be because the higher fibers in the guts increased the obligate anaerobic microbial composition reducing the proportion of facultative anaerobes. The latter are known hosts of inflammation and antibiotic resistance. Additionally, as compared to plant‐based foods, animal‐derived foods have the potential for a higher transfer frequency of ARGs within the human gut^77^. Another study found that long‐term administration of high‐fat diets in mice may impair the efficacy of bactericidal antibiotics by altering the diversity and composition of gut microbiota, and increase bacterial load and antibiotic tolerance toward ciprofloxacin^79^.

### Influence of age on ARG patterns

Based on findings related to gut ARGs in four different age groups (preschool‐aged children, school‐aged children, high school students, and adults), the detected number of ARGs in the human gut microbiome gradually accumulated from childhood (25 gene types) to adulthood (72 gene types), and the ARGs‐type diversity coefficients (Gini‐Simpson index) with age increased from 0.061 to 0.202^80^. Similar findings were observed in healthy young adults (18–45 years), older adults (above 60 years of age), and centenarians up to 109 years of age^81^, ^82^. Interestingly, aging was found to be related to the increasing burdens of most ARGs. However, some ARGs are similarly represented in all age groups, potentially constituting part of the core resistome^82^. The trend seems that as humans are exposed to antibiotics, ARGs, and ARB over their lifetime, they accumulate a higher burden of ARGs and ARB. While some studies have provided valuable information to discuss the age distribution of resistance genes, the nonnegligible impact of other factors, such as the health status of host species, diets, antibiotic administration, and living conditions coexisted in those evaluated systems, thus largely interfering with the exact presentation of the age‐dependent pattern of ARG prevalence. Different from the ARG patterns in humans, calves were rapidly colonized by *Amp* ^r^ *E. coli*, with peak prevalence values observed in 4‐month calves, followed by a declining trend as the calves increased ages (*p* < 0.001)^83^. The age‐dependent pattern of ARB (i.e., *E. coli* and *Salmonella* species) in cattle farms is typical, and young stocks (i.e., preweaned stages) universally possess higher abundances of ARG‐carrying bacteria than old stocks^83^. However, antibiotic treatment did not appear to directly affect the carriage level of calf cohorts, but total levels of *E. coli* did decrease over time, potentially attributing the decrease in resistance to a change in competition in an evolving gut environment or the alteration of the colonization ability of resistant strains.

### Gender and antimicrobial resistance

Females have been found to possess higher levels of certain ARGs, including macrolide‐lincosamide‐streptogramin, levofloxacin, and beta‐lactamase resistance genes^84^, ^85^, ^86^. Women are 27% (prevalence rate ratio, 1.27 ±  0.12) more likely than men to receive an antibiotic prescription in their lifetimes, especially with more macrolides and cephalosporins prescribed to women during 16–54 years of age^87^, indicating that antibiotic use might be a major risk factor for antimicrobial resistance development in women. On the contrary, the prevalence of antimicrobial resistance in stool samples from female children (*n* = 57) in a rural area was significantly lower than that of their male counterparts (*n* = 68) (*p* = 0.043), largely related to infrequent access to antibiotics^88^. Consequently, gender may not directly influence ARG prevalence, but factors such as the antibiotic type used, hormonal changes, and fertility‐related aspects might drive the observed gender differences^87^, ^89^.

### Living conditions associated with increased antimicrobial resistance

Living conditions, including measures relating to the living environment, working conditions, barriers to housing and services, economic status, and proximity to hospitals with high antibiotic prescription rates, are closely associated with antibiotic consumption, further significantly affecting the prevalence of ARGs. For example, rural individuals, particularly women and children, with prolonged exposure to poultry tend to have a higher abundance of ARGs in their gut microbiome^90^, ^91^. There is substantial evidence suggesting high horizontal gene transfer of ARGs within and between households and backyard farms^92^. In a study investigating the impact of working on a swine farm, the abundance of three types of ARGs (beta‐lactamase, aminoglycoside, and tetracycline resistance genes) significantly increased after one month of students' visit^93^. However, another study found that the ARG patterns between women exposed to poultry and those exposed to other animals were no longer significantly different after one year^94^. These results suggest that the composition of ARGs in the gut microbiome is temporarily affected by living conditions and may be continuously adjusted by subsequent environmental and living style changes. Patients infected with *Helicobacter pylori* residing in rural areas had a higher prevalence of genes resistant to metronidazole, clarithromycin, and levofloxacin in their gut microbiome isolates (99.53%) than urban patients (82.88%)^95^. These discrepancies may be related to local hygiene standards and variations in antibiotic prescription rates in local hospitals^96^. Moreover, horses from different continents exhibited variations in the abundance of gut ARGs, with significantly higher levels found in domestic animals compared to feral animals (*p* < 0.05)^97^. Geographical disparities in the incidence of resistance genes in the gut microbiome have also been observed between human populations from different countries^98^.

### Probiotics impact the gut ARG reservoir

Probiotics, defined as “live microorganisms administered for their host health benefits”, are being increasingly utilized as a strategy to improve the gut microbiome^99^. However, the potential that these probiotics contribute to antimicrobial resistance is a growing concern, and it remains controversial whether probiotics promote or inhibit antimicrobial resistance in the gut microbiome^100^. There is evidence to suggest that probiotics not only harbor ARGs but can also acquire them through genetic mutation and horizontal gene transfer^101^, ^102^, ^103^. A range of probiotic bacteria, including *Bifidobacterium*, *Lactobacillus*, *Streptococcus thermophilus*, *Bacillus*, and *Lactobacillus rhamnosus*, have been found to contain ARGs and confer resistance to multiple antibiotics^104^, ^105^. *Bifidobacterium* and *Lactobacillus*, both common constituents of daily diets, have shown resistance to a variety of antibiotics, such as aminoglycosides, quinolones, polypeptides, mupirocin, vancomycin, tetracyclines, chloramphenicol, and lincosamides^100^, ^104^, ^106^, ^107^. Moreover, MGEs, especially conjugative plasmids, have been identified in numerous probiotics, demonstrating their ability to transfer ARGs to pathogenic bacteria and normal gut microorganisms, both in vivo and in vitro^101^, ^102^. This potential for gene transfer underscores the possible pathogenicity of probiotics and warrants further consideration. Conversely, a significant decrease in the detected number and relative abundance of ARGs was observed in the gut microbiome of COVID‐19 patients following probiotic supplementation during treatment^108^. Notably, the probiotics used were detected in 40% of the gut microbiome samples, and the complete removal of beta‐lactamase‐producing *Enterobacteriaceae* from the gut was found to be 2.71 times more effective with probiotics^109^. The effect of probiotics on ARGs in the gut microbiome may be related to their ability to colonize the gut^110^.

## STRATEGIES TO COMBAT GUT ANTIMICROBIAL RESISTANCE

**Figure 3:**
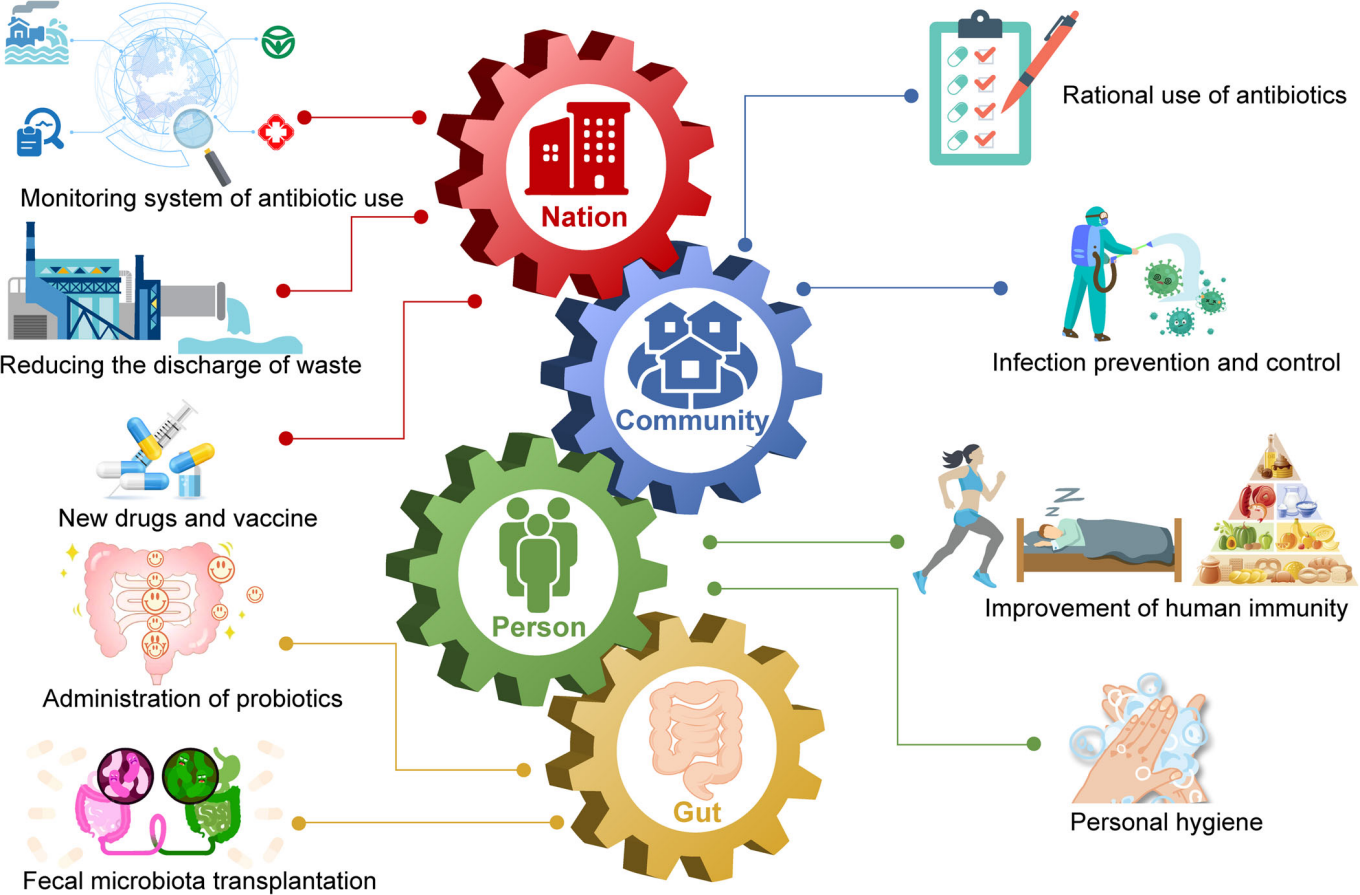
Strategies to combat gut antibiotic resistance from the levels of nation, community, person, and gut management.

The emergence of new risk factors (e.g., lack of enforcement of relevant legislation, poor infection and disease prevention, vague patient clinical information, and drug misuse) in recent years calls for appropriate responses to this escalating crisis of antimicrobial resistance. For these identified risk factors, the following strategies may improve resistance management (Figure 3).

### National strategies to combat antimicrobial resistance

#### Surveillance of antimicrobial resistance

An urgent need exists for the establishment of antimicrobial resistance surveillance and early warning mechanisms within food hygiene, environmental, and sanitation monitoring systems. By utilizing metagenomics‐based sequencing technologies for both known and yet‐to‐emerge pathogen detection, long‐term sampling and monitoring of pathogenic bacteria should be undertaken for antimicrobial resistance analysis^111^. In conjunction with strain research, an information network (e.g., cloud computing, Data Analytics, and Blockchain) should be established with medical institutions, contributing to the prevention of pathogenic infections and guidance for clinical treatment. Monitoring antimicrobial resistance in all relevant settings can greatly enrich the understanding of the dissemination pathways of ARB/ARGs, thereby guiding infection prevention and antibiotic selection^112^. For example, by tracking dynamic changes in the antibiotic resistome and the inflow/outflow ratio of ARB and ARGs during the wastewater treatment process^113^, an integrated overview of ARB and ARG fate in complex wastewater systems can be acquired, thus serving as a guide for the removal of ARB and ARGs.

#### Interventions to reduce the dissemination of antimicrobial resistance

There is a pressing need for stricter legislation to minimize antibiotic usage, including prohibiting the direct discharge of untreated waste/wastewater into the environment. Both human^114^ and animal^115^ feces are key sources of ARB and ARGs in the environment. Measures to reduce ARB during the safe disposal of human and animal waste/wastewater should be implemented, such as improving sanitation, introducing probiotics or nutritional supplements in feed, and vaccinating against common animal diseases^116^. The use of composting technology can decrease the abundance and expression level of ARGs in feces, and studying and promoting optimal composting conditions for reducing ARB should be encouraged^117^. Likewise, pyrolysis technology can be used to turn fecal matter into biochar thereby effectively mitigating environmental dissemination of ARGs while at the same time storing carbon in farmland soils^118^.

#### New antibiotics and vaccines

Recent scientific and technological advancements have opened new avenues to effectively combat antimicrobial resistance. The World Health Organization has called for incentives for new antibiotic and vaccine research and development^119^. Government bodies, research institutes, and regulatory agencies should continue their resources and funding toward the development of new antibiotics. In terms of new antibiotic research and development, efforts should not only aim at discovering new antibacterial agents but also at improving drug efficiencies, such as the combinations of antibiotics with nonantibiotic activity and of different antimicrobials, offering more practical strategies to control and prevent antimicrobial resistance^120^. Unlike antibiotics, vaccines can provide long‐lasting effects on specific strains or diseases within several vaccinations. Vaccines could not only target specific diseases, reduce health care costs, and decrease long‐term complications related to infectious disease but also potentially decrease the emergence of ARGs by reducing the consumption of antibiotics^121^. The proportion of healthcare‐associated infections that are also antimicrobial‐resistant decreases with increased vaccine coverage, particularly for certain combinations of vaccines, pathogens, and antibiotics^122^. Currently, vaccines against infectious agents like *Streptococcus pneumoniae* and *Haemophilus influenzae* have demonstrated a reduction in antimicrobial resistance; however, more research and development are needed for vaccines against antibiotic‐resistant pathogens, such as *Vibrio cholera*, *S. enterica* ser. Typhimurium, *E. coli*, hospital‐acquired infections, and viral agents of pulmonary and diarrheal diseases^123^. Although there are various upcoming difficulties in exploring a viable vaccine, more studies have provided valuable evidence for immune response mediated by the vaccine and antigens that effectively reduce the proliferation of antimicrobial resistance and can pave the way for vaccine discovery and development.

### Antimicrobial resistance control in a clinic or healthcare setting

#### Rational use of antibiotics

The oral delivery of antibiotics has its advantages, such as being convenient, quick, and less likely to cause acute allergic reactions^124^, ^125^. This has led to a strong inclination toward oral antibiotic administration within the medical system. However, for asthma, inhaled medications offered targeted delivery to the site of the disease, lower dosages compared to oral or intravenous routes, reduced systemic absorption, and subsequently decreased risk of adverse effects^126^. The impact of antibiotic delivery routes on antimicrobial resistance should be taken seriously, particularly due to interactions with intestinal microbiota^127^. There is evidence to suggest that oral antibiotic administration, specifically, has a prominent effect on raising the levels of intestinal antimicrobial resistance, and alternative drug administration routes (e.g., injection) may minimize the amplification and development of gut ARGs under the same conditions^128^. More important, the unnecessary prescription and dispersion of antibiotics to patients/farmers should be banned. In the human sector, rational use of antibiotics must be considered an issue of patient safety, as antibiotic use can have direct negative side effects on the individual consumer^129^. In the food animal sector, governments are recommended to provide appropriate regulations on the authorization, manufacture, distribution, and use of veterinary products through their veterinary legislation^130^.

#### Prevention of healthcare‐acquired infections

Presently, *Clostridioides difficile* infection has been viewed as one of the leading causes of antibiotic‐associated diarrhea in hospitalized patients^131^. An obvious reduction in antibiotic consumption, due to antibiotic stewardship programs, resulted in a decrease in the incidence of healthcare facility‐onset *C. difficile* infection^132^. This indicates that preventive measures against infection should be taken to curb the spread of pathogens, including resistant ones, within clinics and healthcare facilities. Such measures can prevent further infections and limit the spread of antimicrobial resistance^133^. In addition, past evidence has shown that hospital infections, including serious ARB infections, can be drastically reduced by improving disinfection and sterilization procedures and minimizing cross‐infection^134^. Certainly, regular maintenance must be performed and documented, including the date of service, location, name and signature of the controller, and biological testing records.

### Personal measures to alleviate antimicrobial resistance burden

#### Improvement of human immunity

Boosting human immunity can guard against pathogenic bacteria invasion, thereby reducing antibiotic consumption. Regular exercise^135^, adequate sleep^136^, and a balanced nutrient intake^137^ can enhance human immunity. A person's long‐term diet greatly influences the composition, activity, and dynamics of their gut microbiome^138^. A balanced diet, providing a moderate intake of macronutrients (proteins, fats, and carbohydrates) and micronutrients (minerals and vitamins), supports overall health and well‐being and impedes the emergence and proliferation of resistant bacteria^139^. Additionally, to effectively combat the spread of antibiotic resistance by dietary means, individuals should prepare food hygienically and obey these five rules (i.e., use safe water and raw materials, cook thoroughly, separate raw and cooked, keep food at a safe temperature, and keep the entire process clean) and choose food ingredients that have been produced without the use of antibiotics as much as possible^140^.

#### Concern for personal hygiene

Maintaining personal hygiene and cleanliness remains a vital strategy for good health^141^. Good hygiene facilitates the fight against antimicrobial resistance through infection prevention from the polluted environment or via frequent person‐to‐person interactions, thus reducing the need for antibiotic prescribing^142^. Preventing infections by regularly washing hands, preparing food hygienically, and avoiding close contact with sick people, are important to address the crisis of antibiotic resistance. Currently, using alcohol‐based hand rubs can prevent the spread of germs, including those that are resistant to antibiotics^143^, thereby inhibiting the dissemination of antimicrobial resistance. The use of personal care products containing antimicrobial chemicals, such as triclosan, triclocarban, and benzalkonium chloride, can help reduce disease transmission in most households, despite concerns about potential hazards and the risk of emerging antimicrobial resistance^141^. Certainly, maintaining strict environmental hygiene is an essential component of preventing and controlling antimicrobial‐resistant infections. Decreasing the load of contagious microorganisms by multi‐layered processes that contain physical removal (i.e., wiping and ventilating) and chemical sterilization (i.e., detergents and chemical disinfectants) is conducive to reducing the possibility of the transfer of infectious germs from object to person, thus lowering the risks of ARG spread^144^.

### Microbiota‐mediated protection against antimicrobial resistance

#### Administration of probiotics

There is growing interest in controlling intestinal microbiota resistance through the ingestion of probiotics or the transplantation of exogenous bacteria. However, it is important to carefully evaluate these interventions before practical applications to avoid introducing new antimicrobial resistance or other unknown risk factors. Growing evidence has shown the effects of probiotic consumption on their host health. According to the selection criteria for novel probiotic candidates from the Food and Agriculture Organization, probiotics are well‐defined safe gut commensal microbes that should meet the requirements of safety assessment (including antibiotic resistance), functional and technological criteria, and desirable physiological criteria. Commercially available probiotics are often derived from a limited range of bacteria and yeast, most commonly *Lactobacillus* spp. and *Bifidobacterium* spp. In the United States, these bacteria have been granted “Generally Regarded as Safe” status, while in Europe, they have received a Qualified Presumption of Safety status from the European Food Safety Authority, though they are not formally approved for health effects^145^.

#### Fecal microbiota transplantation

Fecal microbiota transplant (FMT) is a therapeutic approach where stool from a healthy donor is introduced into a patient's digestive tract via nasogastric, nasoduodenal, enema, nasojejunal routes, or colonoscopy^146^. This procedure is a viable treatment for patients with recurrent *Clostridium difficile* infection, ulcerative colitis, and other nongastrointestinal disorders^147^. Oral capsules of FMT have also been developed^148^. Unlike probiotics, FMT aims to introduce a stable and complete community of bacteria into the gut, which can help repair or replace an altered native microbiota and confer a health benefit^145^. The positive effects of FMT on host health encouraged scientists to eliminate ARB colonization by replacing a healthy gut microbiome. In this regard, the FMT method has been applied in patients with recurrent *C. difficile* infection, and effectively reduced the carriage of multiple ARGs^149^. Recently, a randomized, controlled trial was conducted to compare the efficacy, safety, and strain dynamics of multidrug‐resistant organisms eradication after FMT, resulting in fast decolonization of multidrug‐resistant organisms and protecting recipients from recurrent infection^150^. Therefore, FMT offers a potentially effective alternative to ARB decolonization in the gut.

## CONCLUSIONS AND FUTURE PROSPECTS

The gut microbiota is a complex ecosystem intimately involved in numerous physiological functions and is widely recognized as an important reservoir for ARGs. Even though gut microbes vary in their mechanisms for acquiring, maintaining, and disseminating ARGs, frequent horizontal and vertical transfers to receptive cells, including pathogens, contribute significantly to the propagation of antimicrobial resistance. The diversity and abundance of the gut resistome are dynamic, with factors such as consumption of antibiotics and probiotics, diet, host physiological status, and lifestyle, all influencing the microbial community composition and structure of the gut resistome. Given the intricate web of interactions among the host, gut microbiota, and factors contributing to antimicrobial resistance in real‐world scenarios, it is impossible to untangle this knot using a simple, “one‐size‐fits‐all” strategy. The One Health concept promotes transdisciplinary collaboration to objectively dissect this multidimensional problem, revealing interconnections and interdependencies among microbes, animals, plants, humans, and ecosystems from both micro and macro perspectives. More importantly, the One Health approach provides an international platform for communication and cooperation among physicians, pharmacists, nurses, veterinarians, farmers, ecologists, and other scientific, health, and environment‐related professionals to tackle antimicrobial resistance. Thus, the global threat of antimicrobial resistance necessitates interdisciplinary and collaborative efforts across all domains within the One Health framework. To effectively mitigate the threat and potential harm of acquired antibiotic resistance, we propose a special focus on four key areas in the future: (1) source tracking of ARGs, (2) mechanisms of ARG dissemination, (3) ARG reduction strategies, and (4) risk evaluation associated with the ARGs.

(1) *Source tracking of ARGs*: Due to the widespread antimicrobial resistance as one of the most public health concerns, accurate quantitative source tracking of environmental ARG pollution is urgently needed. However, there are inherent limitations existing in quantitative approaches for ARG detection of the environmental component. One challenge for culture‐based methods is that over 99% of microbial species have not been cultured or recognized, which enormously restricts our exploration of this uncharacterized microbial world^151^. Although these drawbacks have been overcome by molecular‐based methods, inadequate dedicated primers for ARG detection and amplification errors due to various biases and false negative/positive results slow the progress of molecular‐based methods to track ARG contamination^152^. Recent advances in targeted high‐throughput sequencing technologies offer novel sequence‐based analysis, broadening our insights into the functional‐gene‐focused repertoire of a sample. For detection and annotation of ARGs in environmental samples, the obtained metagenomic reads can be filtered and processed in a reference ARG database (e.g., the Comprehensive Antibiotic Resistance Database) that contains sequences of known ARGs^153^. Enriching the understanding and identification of unknown ARG targets remains a big challenge in source tracking studies, and machine‐learning classification approaches will be an aid in sorting and predicting quantitative information embedded in this big data era. Considering the long‐proposed significant correlation between bacterial structure and ARG prevalence, and the predictable variation of ARG patterns under certain spatiotemporal variability and environmental conditions, an effective source tracking system would facilitate the accurate ARG pollution assessment and design modulatory strategies at relevant hot spots. Specifically, coupling traditional (e.g., culture‐based and PCR‐based methods) and emerging (e.g., metagenomic analysis tools and machine‐learning classification) approaches to form an integrative source tracking framework will have great implications, including the risk ranking of different ARG sources and subsequent source‐sink relationship identification.
(2) *Mechanisms of ARG dissemination*: While numerous studies have primarily focused on the overall prevalence of ARGs, in‐depth research into the driving mechanisms and the extent of mobile ARGs via resistance‐associated MGEs is lacking. Comprehensive characterization of resistance‐associated MGEs, the fitness landscape associated with MGEs encoding ARGs, their migration behaviors, and the enhancement of bioinformatics algorithms (including annotation and classification of MGEs), may aid in better interpreting the interconnected relationships between pathogens and commensal organisms in the evolution and dissemination of ARGs.
(3) *ARG reduction strategies*: The development of appropriate strategies to combat persistent antimicrobial resistance is urgently needed, given the potential impacts on morbidity, socioeconomic losses, and mortality associated with antimicrobial resistance. First, a broad ensemble of political and regulatory actions are necessary such as legislation formulation for preventing the inappropriate introduction and discharge of veterinary medicinal products, policy guidance on minimizing ARG release into different environmental compartments, and codes of good practice in supporting ARG prevalence reduction by respective scientific pretreatments. Then some specific approaches can be recommended. For example, emerging alternative therapies, such as antibiotic combinations, bacteriophage therapy, the use of antibiotic adjuvants, monoclonal antibodies and vaccines, and fecal microbiota transplantation, can bypass existing resistance mechanisms to curb the spread of ARGs. These promising methods may offer significant potential for ARG control. However, a thorough assessment and further development are needed before they can be applied broadly.
(4) *Risk evaluation associated with the ARGs*: While the perception of antimicrobial resistance as a global threat is widespread, the task of evaluating their relative health risks remains challenging. The health risks of antimicrobial resistance can initially be understood as the estimated infectious diseases, and associated deaths caused by ARB, under the selection pressure of environmental antibiotic residues. As ARB acts as the most critical link in assessing antimicrobial resistance risks, the occurrence probability can be predicted based on the assumed quantitative dose–response relationship between antibiotic dose and the likelihood of infectious events. Therefore, the identification of morbidity and mortality attributable to specific pathogens resistant to antibiotics could be used to evaluate the health risks. However, the data on antibiotic residues, pathogenic infection events, and antimicrobial resistance risks are currently insufficient to precisely construct an experimental evaluation model. There is a pressing need to build a comprehensive database to fill these data gaps.
